# Limb Immobilization Induces a Coordinate Down-Regulation of Mitochondrial and Other Metabolic Pathways in Men and Women

**DOI:** 10.1371/journal.pone.0006518

**Published:** 2009-08-05

**Authors:** Arkan Abadi, Elisa I. Glover, Robert J. Isfort, Sandeep Raha, Adeel Safdar, Nobuo Yasuda, Jan J. Kaczor, Simon Melov, Alan Hubbard, Xiaoyan Qu, Stuart M. Phillips, Mark Tarnopolsky

**Affiliations:** 1 Department of Pediatrics & Medicine, McMaster University, Hamilton, Ontario, Canada; 2 Procter & Gamble Company, Mason, Ohio, United States of America; 3 Graduate School of Medicine, Juntendo University, Inba, Japan; 4 School of Medicine, Debinki 1, Gdansk, Poland; 5 Buck Institute for Age Research, Novato, California, United States of America; 6 School of Public Health, University of California, Berkeley, California, United States of America; 7 Department of Kinesiology, McMaster University, Hamilton, Ontario, Canada; Texas A&M University, United States of America

## Abstract

Advancements in animal models and cell culture techniques have been invaluable in the elucidation of the molecular mechanisms that regulate muscle atrophy. However, few studies have examined muscle atrophy in humans using modern experimental techniques. The purpose of this study was to examine changes in global gene transcription during immobilization-induced muscle atrophy in humans and then explore the effects of the most prominent transcriptional alterations on protein expression and function. Healthy men and women (N = 24) were subjected to two weeks of unilateral limb immobilization, with muscle biopsies obtained before, after 48 hours (48 H) and 14 days (14 D) of immobilization. Muscle cross sectional area (∼5%) and strength (10–20%) were significantly reduced in men and women (∼5% and 10–20%, respectively) after 14 D of immobilization. Micro-array analyses of total RNA extracted from biopsy samples at 48 H and 14 D uncovered 575 and 3,128 probes, respectively, which were significantly altered during immobilization. As a group, genes involved in mitochondrial bioenergetics and carbohydrate metabolism were predominant features at both 48 H and 14 D, with genes involved in protein synthesis and degradation significantly down-regulated and up-regulated, respectively, at 14 D of muscle atrophy. There was also a significant decrease in the protein content of mitochondrial cytochrome *c* oxidase, and the enzyme activity of cytochrome *c* oxidase and citrate synthase after 14 D of immobilization. Furthermore, protein ubiquitination was significantly increased at 48 H but not 14 D of immobilization. These results suggest that transcriptional and post-transcriptional suppression of mitochondrial processes is sustained throughout 14 D of immobilization, while protein ubiquitination plays an early but transient role in muscle atrophy following short-term immobilization in humans.

## Introduction

Skeletal muscle atrophy is associated with bed rest, corticosteroid use, denervation, chronic renal failure, limb immobilization, neuromuscular disorders, sarcopenia of aging, and arthritis [1]–[8]. Irrespective of the underlying cause of atrophy, reduced muscle activation/contractile activity (hypodynamia) is an invariant feature. Recently, strong interest has focused on characterizing the fundamental molecular mechanism(s) underlying muscle atrophy and numerous cellular processes are known to coalesce into the overall atrophy phenotype. These alterations include decreased protein synthesis, increased protein degradation, and suppression of bioenergetic pathways associated with mitochondrial function, and increased oxidative stress [9], [10].

Upstream triggers that initiate atrophy are poorly understood and may vary depending on the pathological context; however, animal data suggests that disparate atrophic stimuli converge on the activation of protein degradation, particularly the ubiquitin (Ub)-26S proteasomal pathway [1], [11]–[14]. Two novel Ub-protein ligases, atrogin-1 (muscle atrophy F-box protein) and muscle ring-finger protein (MuRF-1), are consistently up-regulated in murine models of muscle atrophy, and are thought to ubiquitinate both regulatory (e.g. calcineurin and MyoD) and structural (e.g. myosin and troponin I) proteins, thus directing the specific degradation of proteins during muscle atrophy [1], [12], [13], [15]–[21]. While much progress has been made towards delineating the underlying functional alterations and signaling pathways that mediate muscle atrophy in animal models, few studies have examined muscle atrophy in humans. Early reports concerning protein turnover in humans demonstrated that mixed muscle protein synthesis rates decline during muscle atrophy while protein degradation rates appear unchanged [22]–[25]. This was confirmed in a recent study in which the rate of myofibrillar protein synthesis decreased by ∼50% following 10 d of unilateral limb suspension (ULS) in human subjects [26]. These studies have emphasized the suppression of protein synthesis during atrophy in human muscle, which contrasts with studies in murine models that point primarily towards increased protein degradation [12]–[14]. However, one recent study found that myofibrillar protein degradation was increased in humans as early as 72 h following ULS [27]. In addition, the expression of atrogin-1 and MuRF-1 during muscle atrophy in humans is contentious, with some studies showing increased atrogin-1 and MuRF-1 mRNA, but not others [26], [28]–[31].

Global micro-array analysis has emerged as a powerful experimental tool to investigate multiple cellular pathways simultaneously; however, the relatively large sample size cost needed to reach the full potential of this technique has limited its application to the study of human muscle atrophy. The purpose of this study was to examine changes in global gene transcription during immobilization-induced muscle atrophy in humans and then explore the effects of the most prominent transcriptional alterations on protein expression and function in a relatively large cohort of men and women. Overall, the transcriptional suppression of bioenergetic and mitochondrial genes dominated the immobilization-induced transcriptome and was evident as early as 48 hours (48 H) following immobilization. These transcriptional changes were accompanied by declines in both the protein level and enzymatic activity of several mitochondrial proteins following 14 days (14 D) of immobilization. In addition, atrogin-1 and MuRF-1 mRNA was significantly up-regulated early during the progression of muscle atrophy and protein ubiquitination was increased following 48 H of immobilization but not 14 D. Lastly, mTOR phosphorylation decreased significantly following 48 H of immobilization but not 14 D.

## Methods

This data was collected during an immobilization study where the subject characteristics and design were described previously [32]. Muscle sufficient for the gene microarray, RT-PCR, protein, and enzyme activity measurements were available for a total of 12 men and 12 women.

### Subjects

Recreationally active, non-smoking, healthy men (N = 12) and women (N = 12) participated in the trial. Demographics included: age (men = 20.5±1.7; women = 21.1±2.7 y), height (men = 178.4±5.4; women = 164.3±5.5 cm), and body mass index (men = 24.3±3.5; women = 22.8±2.2) (Table 1). All of the women were eumenorrheic and ∼50% were tested in each phase of the menstrual cycle. The study was approved by McMaster University and the Research Ethics Board of the Hamilton Health Sciences. The study was explained in detail to all subjects and they provided written informed consent before participation.

**Table 1:** Subject Characteristics.

	Men	(n = 12)		Women	(n = 12)	
	PRE	48 H	14 D	PRE	48 H	14 D
***Subject Characteristics***						
Age (y)	20.8±2.0			21.1±2.7		
Height (cm)	177.9±7.7			164.3±5.5*		
Body Mass (kg)	76.1±11.0	76.8±11.3	76.6±11.8	61.8±9.1*	61.9±9.3*	61.6±9.5*
Lean Body Mass (kg)	62.0±7.6	61.8±7.7	62.0±8.0	44.8±5.3*	44.5±5.5*	44.6±5.5*
***Hormones***						
Total Testosterone (nmol•l^−1^)	14.1±2.4	14.0±3.1	14.1±4.1	0.8±0.3*	0.7±0.4*	0.7±0.2*
Estradiol (pmol•l^−1^)	105±23	103±31	101±30	153±186	159±153	115±82
Cortisol (nmol•l^−1^)	598±164	501±184	466±131	732±223	656±144* †	640±240* †
***Muscle Characteristics***						
CSA Total Quadriceps Femoris (cm^2^)	81.9±12.0	N/A	76.9±11.56†	57.3±7.4*	N/A	54.0±9.0* †
Concentric Slow Knee Ext. (Nm)	218±36	204±29	194±28†	146±34*	129±31*	115±29* †
Isometric Knee Strength (Nm)	253±36	248±37	232±33	159±40*	139±41*	123±42* †

***Notes*:** CSA = cross sectional area. Cortisol was analyzed using men n = 11 and women n = 9, and total testosterone was analyzed using women n = 11. Values are means ± SD.

* indicates a difference between women and men with a p value <0.05.

† indicates a change over time with a p value of <0.05.

### Experimental procedure

Subjects had a randomly assigned leg immobilized using a knee brace (epX Knee Control Plus, Smith Orthopedics, Topeka, KS) and were provided with walking crutches such that there was no weight bearing on the immobilized leg. The study consisted of three testing sessions termed; PRE (5 d before immobilization), 48 H (48 hours after immobilization) and 14 D (14 days after immobilization). The subjects did not exercise for at least 3 d before the first testing session or immobilization. All testing sessions were identical and occurred in the morning following an overnight fast, with consumption of a defined formula diet (500 mL; Boost®, Novartis Medical Health, Inc., NY) 2 h prior to the muscle biopsy. Testing included dual X-ray absorptiometry (DEXA), magnetic resonance imaging (MRI), muscle strength, muscle biopsy and blood sampling, with full details described previously [32]–[36]. MRI was completed in a 1.5 Tesla scanner with superconducting magnets (Symphony Quantum, Siemens, Erlangen, Germany) to determine the cross-sectional area (CSA) of the *vastus* muscles (*vastus lateralis*, *vastus intermedius* and *vastus medialis*) and *rectus femoris* [32].

Muscle biopsies were obtained from the *vastus lateralis* muscle of the immobilized leg under local anesthesia (2% lidocaine) with a modified Bergström biopsy needle with manual suction. Incisions were made at randomly chosen proximal, distal or mid (15 cm proximal to lateral joint space) sites of the *vastus lateralis* separated by ∼3 cm. There were 7 d between biopsies from PRE to 48 H and 12 d from 48 H to 14 D. Each sample was immediately dissected of fat and connective tissue. Samples were divided into three separate pieces, placed into RNAase free tubes, plunged into liquid nitrogen, and stored at −80°C. If there was sufficient muscle tissue remaining after the three primary allocations, we embedded the muscle in optimal cutting temperature medium and plunged it in to *N*-methylbutane chilled in liquid nitrogen for histological fiber analysis using ATPase staining as previously described by our group [32]. Muscle strength testing was conducted at each session using a dynamometer (Biodex-System 3, Biodex Medical Systems Inc., New York) to evaluate the relative changes in maximal force generating capacity over the time course of immobilization.

### RNA extraction

Total RNA was extracted from muscle using TRIzol® Reagent (Invitrogen Canada) following the manufacturer's instructions. The concentration and purity of the RNA was spectrophotometrically determined by measuring the absorbance at 260 (OD_260_) and 280 (OD_280_) with an OD_260_/OD_280_ ratio of ∼1.8 indicating high quality RNA. The integrity of RNA samples were assessed by RNA agarose-formaldehyde gel electrophoresis and evaluating the ratio of 28S to 18S rRNA bands.

### Gene Expression Analysis

Global changes in gene expression were examined using the HG U133 plus gene micro-array (Affymetrix, Santa Clara, CA) containing 54,675 human probe sequences. The background correction, normalization and derivation of expression measures were based on the Affymetrix signal (MAS 5.0 algorithm). Data quality and outliers were checked using exploratory statistical tools, such as summary statistics, pair plots, and Principal Component Analysis. The micro-array data discussed in this publication have been deposited in NCBI's Gene Expression Omnibus [37] and are accessible through GEO Series accession number GSE14901 (http://www.ncbi.nlm.nih.gov/geo/query/acc.cgi?acc=GSE14901).

Gene filtering was performed using standard multiple testing procedures and clustering, based on packages available in Bioconductor (www.bioconductor.org). First, expression of genes at 48 H and 14 D after immobilization was compared to baseline (PRE) using standard paired t-tests to define the early and late transcriptional response to immobilization, respectively. For each of these sets, we choose “significantly” differentially expressed genes based on a false discovery rate (FDR) q-value <0.05. 576 genes and 3,128 genes had significantly altered expression levels (FDR q-value <0.05) at 48 H and 14 D, respectively (Table 2). To ascertain which transcriptional changes were specifically associated with muscle atrophy, a trend test (using simple linear regression of Log_2_ expression versus change in cross sectional area (CSA)) was conducted as the criterion for differentially expressed genes. Only 239 genes were found to have an FDR q-value <0.05, in the analysis of atrophy-associated genes.

**Table 2:** Summary of micro-array analyses.

*Analysis*	*Up-regulated genes*	*Down-regulated genes*	*Total*	*Unknown*
Early (48 H) genes	243	332	575	26%
Late (14 D) genes	1,514	1,614	3128	30%
Atrophy-associated genes	86	153	239	26%

***Notes*:** Listed are the number of probes in each micro-array analysis which were significantly (FDR q-value <0.05) altered by immobilization in human muscle.

Public resources as well as commercially available tools were used to annotate the expressed genes, including Netaffx (http://www.affymetrix.com/index.affx) and GeneCards (*Weizmann Institute of Science & Crown Human Genome Center, URL:* http://bioinfo.weizmann.ac.il). For those uncharacterized transcriptional probes or ESTs, BLAST searches against NCBI non-redundant databases were conducted. Gene Ontology (http://www.geneontology.org) was coupled to comprehensive literature research to organize genes into 18 broad functional groups which include; mitochondria (oxidative phosphorylation, tricarboxylic acid cycle, fatty acid metabolism, regulatory, transport, and metabolism), carbohydrate metabolism, metabolic (referring to non-bioenergetic metabolic pathways), signaling and transcription factors, oxidative stress, redox, chaperones, metallothioneins, secreted factors, biosynthesis, RNA synthesis and maturation, protein synthesis, DNA regulation (including repair enzymes and chromatin organization factors), protein degradation, cytoskeleton, extracellular matrix, cell surface (receptors, markers, channels and transporters), and organelle (referring to non-mitochondrial organelles and including vesicle trafficking, Golgi apparatus, endoplasmic reticulum, lysosomes). Once genes were organized into functional categories, χ^2^ analyses were conducted in which the actual number of up- and down-regulated genes within each functional category was compared to the expected number of up- and down-regulated genes. The expected number of up- and down-regulated genes was calculated by dividing the total number of genes within each category by the total number of up- and down-regulated genes, respectively. Functional categories with P≤0.05 were considered to be significantly over- or under-represented during immobilization.

In addition, genes that had an FDR q-value <0.05 were processed using the hierarchical clustering method (HOPACH) [38] to find groups of similarly expressed genes across arrays and examine the ordered distance matrices returned by this clustering algorithm. In the analysis of late transcriptional changes following immobilization (PRE versus 14 D), genes with an FDR q-value <0.05 were ranked by FDR and only the top 1000 genes were considered in the HOPACH cluster analysis. The gene content of clusters varied considerably making an assessment of gene cluster and function relationships not immediately obvious. To examine the relationship between gene clustering and gene function, clusters were grouped directionally (i.e. up-regulated clusters and down-regulated clusters were separated) and the distribution of genes within a given functional category across clusters were examined using χ^2^ analyses. Thus the actual gene content of individual clusters within each functional category was compared to the expected number of genes within said cluster and category. The expected gene content of a cluster within a category was calculated as the product of the total number of genes within the cluster and the proportion of genes within that functional category (i.e. total number of up-/down-regulated genes within the category divided by the total number of up-/down-regulated genes). Functional categories in which genes were unevenly distributed over clusters (P≤0.05) were considered to show significant clustering along functional lines. Note that because some clusters contained very few genes within a given functional category, only functional categories that contained a total number of genes (N) greater than the total number of clusters (N_C_) plus 2 (i.e. N>N_C_ +2) were considered for partitioning analysis (χ^2^).

#### qRT-PCR

1 µg of RNA, DNase treated (DNA-free™, Ambion Inc, Austin TX) was converted to cDNA using the 1st Strand cDNA Synthesis Kit for RT-PCR (AMV) (Roche Applied Science, Laval PQ). Briefly, the reaction volume of 20 µL for RT contained: 1x reaction buffer, 5 mM MgCl_2_, 1 mM dNTP mixture, 3.2 µg of random primer p(dN)_6_, 50 U of RNase inhibitor, 0.8 µL of AMV reverse transcriptase, and 1 µg of DNA-free RNA. RT was performed in a thermal cycler (Applied Biosystems, Foster City, CA): 25°C for 10 min, 42°C for 60 min, 99°C for 5 min, and 4°C for 5 min. All RNA samples were converted to cDNA together to minimize technical variability. Negative controls (no RNA or no reverse transcriptase enzyme) were run simultaneously with samples to control for RNA and genomic DNA contamination.

The primers for real-time PCR were designed using Primer3 software (http://frodo.wi.mit.edu/, Whitehead Institute for Biomedical Research) and their thermodynamic specificity was determined using BLAST sequence alignments (NCBI) and Oligo analyzer software (Integrated DNA Technologies, Coralville IA). Real-time PCR was performed using an iCycler® real-time PCR system (Bio-Rad Laboratories, CA) using SYBR® Green 1 chemistry (iQ SYBR® Green Supermix, Bio-Rad Laboratories, CA) according to the manufacturer's instructions. Briefly, the reaction volume of 25 µL for PCR contained: 1x iQ SYBR® Green Supermix, forward and reverse primers, 10 ng cDNA template. The application was performed for 1 cycle (50°C for 2 min, 95°C 10 for min) followed by 40 cycles (95°C for 15 s, 60°C for 60 s) and the β2-microglobulin signal was used as a housekeeping gene to normalize C_T_ values. All samples were run in duplicate simultaneously with RNA- and RT-negative controls. In addition, the melting point dissociation curve generated by the instrument was also used to confirm the specificity of the amplified product.

### Protein content

The level of phospho-mTOR, mTOR, Ub, CS, COX subunit II (mitochondrial encoded) and COX subunit IV (nuclear encoded) were quantified using Western blotting, as previously reported by our group [39]. Briefly, 10 µg of 600 *g* muscle homogenate was separated using 7–12.5% PAGE. Phospho-mTOR, mTOR (rabbit monoclonal, Cell Signaling, Danvers, MA), Ub (rabbit polyclonal, Biomol, Plymouth Meeting, PA), CS (rabbit polyclonal, a kind gift from Dr. Brian Robinson, Hospital for Sick Children, Toronto ON), COX subunit II, and COX subunit IV (mouse monoclonal, Mitosciences, Eugene OR) antibodies were used to probe proteins transferred onto PVDF or nitrocellulose membranes, which were then developed and quantified using the ImageJ software package (NIH, Bethesda, MA, USA).

### Enzyme analysis

We measured the maximal rate of enzyme activity for citrate synthase (CS) and cytochrome *c* oxidase (COX) using a Cary BIO 300 spectrophotometer (Varian Canada, Toronto) at 30°C, as previously described by our group [39]. Briefly, CS activity was measured by adding 10 µL of supernatant to a medium containing 50 mM Tris-HCl buffer +5 mM EDTA at pH 8.1 with 0.1% Triton X-100, 0.1 mM dithionitrobenzoic acid (DTNB) and 0.1 mM acetyl CoA, The enzyme reaction was started with addition of 0.5 mM oxaloacetate. Absorbance was recorded at 412 nm for 3 min. All of the samples were analyzed in duplicate and expressed as nmol/min/mg protein. COX activity was determined as previously described by our group [39], [40] with minor modifications. Stock cytochrome *c* (oxidized) was reduced with sodium ascorbate in 10 mM phosphate buffer at pH 7.0. 10 µL of a 600 g supernatant was added to 970 µl of 50 mM phosphate buffer at 7.4. The reaction was initiated with 20 µL of reduced cytochrome *c* and monitored at 550 nm for 3 min. The protein content of the 600 g supernatant fraction was determined according to Lowry et al [41]. All of the samples were analyzed in duplicate and the COX activity was expressed as nmol of cytochrome *c* oxidized/min/mg total protein using a molar extinction coefficient of 18.9 mM^−1^cm^−1^. The intra-assay coefficient of variance for the enzyme activity assays was less than 10%.

### Statistical Analysis for non-micro-array studies

An independent t-test was applied to assess significant sex-specific demographic differences. A two-way (gender and time) analysis of variance (ANOVA) with mixed design was performed to test for significant differences in muscle strength and muscle CSA using a computerized statistical package (Statistica V5.1, Statsoft, Tulsa, OK). Unpaired Student t-tests were used to test for differences between groups in all other dependent variables (Statistica 5.0, Statsoft, Tulsa, OK). Linear regression analysis was used to correlate the mRNA transcript abundance levels between the gene micro-array and the qRT-PCR analyses. Statistical significance was established at P≤0.05. Data are presented as the means ± standard deviation (SD).

## Results

### Muscle strength and CSA of quadriceps

Both men and women showed significant decreases in absolute peak torque (Nm) following 14 D (men, 6.3±4.4%, P<0.001; women, 23.0±6.1%, P<0.01), with women showing greater strength loss as compared to men (P<0.05) (Table 1). Men and women showed significant and nearly identical decreases in total muscle CSA after 14 D of immobilization (men = 5.3±1.4%; women = 6.2±1.5%, P<0.05), with men showing a larger CSA of total quadriceps femoris (P<0.001) compared to women at all times (Table 1). In addition, men (N = 8) and women (N = 6) showed significant (P<0.05) decreases in type-I muscle fiber CSA following 14 D of immobilization, but only women showed a significant (P<0.05) decrease in type-IIa fiber CSA, and no significant changes in type-IIx fiber CSA were detected in either sex.

### Global gene array analysis

**Figure 1:**
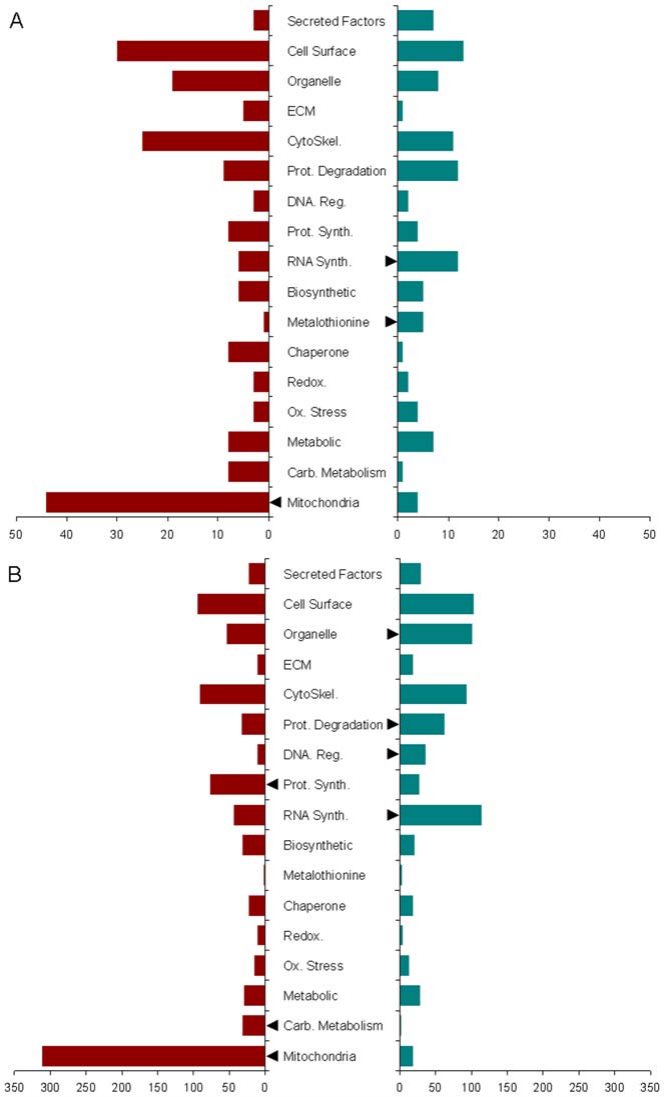
Early (A) and late (B) transcriptional changes during immobilization-induced muscle atrophy. (A) Two-time point comparisons of Log_2_ probe signal between the PRE and 48 H time points were conducted and significant differences were assessed using standard paired T-Tests. Genes with FDR q-value <0.05 were considered to be significantly altered. Genes were then organized along functional lines and the number of genes in each category was plotted. χ^2^ analyses comparing actual versus expected number of genes within each category (as detailed in the Materials and Methods) were conducted to determine whether gene categories were significantly (P≤0.05) up- or down-regulated at the early (48 H) time-point following immobilization. (B) Two-time point comparisons between PRE and 14 D probe signals were conducted as in (A) to identify gene categories that were up- or down-regulated as part of the late (14 D) transcriptional response to immobilization. Red bars are down-regulated genes whereas green bars are up-regulated genes. Arrowheads indicate gene categories that were significantly up- or down-regulated. Gene categories representing fatty acid and carbohydrate metabolism, and mitochondrial function, including oxidative phosphorylation, β-oxidation, and mitochondrial regulation, were significantly down-regulated at both 48 H and 14 D of immobilization. In addition, the gene category representing mRNA synthesis and maturation (e.g. splicing, polyadenelation, 5′ cap binding proteins) was significantly up-regulated at both 48 H and 14 D. The gene category representing metallothioneins was up-regulated at 48 H but not 14 D, while gene categories representing proteases and DNA regulation (e.g. repair enzymes and chromatin remodeling proteins) were up-regulated at 14 D but not 48 H. Lastly, the gene category representing protein synthesis was down-regulated at 14 D but not at 48 H. ECM: extracellular matrix; Prot. Degradation: protein degradation; DNA. Reg.: DNA regulatory factors; RNA Synth.: RNA synthesis and maturation; Ox. Stress: oxidative stress; Carb. Metabolism: carbohydrate metabolism.

Total RNA was obtained from 24 subjects at three time points (N = non-pooled 72 samples) and examined using gene micro-arrays (Affymetrix HG U133plus). Simple two-time point comparisons in Log_2_ expression between PRE versus 48 H, and PRE versus 14 D were conducted to define the early and late transcriptional response to immobilization, respectively. In addition, an analysis was conducted to examine probes whose signal alteration was significantly associated with muscle atrophy. To this end, a linear regression of change in Log_2_ expression versus change in muscle CSA was used in a trend test to identify atrophy-associated genes. Table 2 describes the micro-array analyses and Table S1 contains the full list of significantly (FDR q-value <0.05) of altered probes. The genes corresponding to these probes were organized into functional categories, and the number of up-regulated and down-regulated genes within each functional category was then compared to determine whether categories were generally up- or down-regulated during muscle atrophy (Figure 1 and S1). Importantly, all transcriptome changes were identical in both men and women, and an analysis of transcriptional alterations that were associated with strength decline did not yield any significantly altered probes.

The salient feature of all the bioinformatic analyses was the transcriptional suppression of genes involved in cellular bioenergetics. Gene categories representing carbohydrate metabolism and mitochondrial function; including oxidative phosphorylation, β-oxidation, and mitochondrial regulation; were consistently down-regulated (Figures 1 and S1). 73% of all mRNA species that showed significant transcriptional changes by 48 H were also significantly altered at 14 D. Only 155 probes were altered by 48 H but not at 14 D (Table S2). Thus, many of the mRNA changes were initiated by 48 H progressed through to 14 D of immobilization. Of note, functional categories representing protein synthesis and protein degradation were only significantly altered by 14 D of immobilization (down- and up-regulated, respectively). Other functional groups were also significantly up-regulated or down-regulated in specific analyses but only the bioenergetics gene category was significantly down-regulated in all micro-array analyses (Figures 1 and S1). These findings suggest that the transcriptional suppression of bioenergetics is initiated early during the progression of immobilization-induced muscle atrophy and maintained for the duration of the immobilization period, while transcriptional activation of protein degradation and the suppression of protein synthesis occur later (14 D) (Figures 1).

To further explore patterns in the immobilization-induced transcriptome, probes were organized into clusters (HOPACH) according to commonalities in their transcriptional profiles. To examine the relationship between gene clusters and gene function, the distribution of HOPACH clusters into functional categories was evaluated. Typically genes that were significantly enriched within individual clusters partitioned unevenly across different functional categories (Figure S2, S3 and S4). Thus gene categories corresponding to components of bioenergetics, mitochondria, and the cytoskeleton segregated into separate clusters in all micro-array analyses, while protein synthesis and protein degradation segregated into separate clusters only at 14 D (Figure S2, S3 and S4). This robust relationship between gene clusters and gene functions indicates a coordinate regulation of cellular pathways at the transcriptional level. Interestingly, PGC-1α, the master regulator of mitochondrial biogenesis, was down-regulated at both 48 H and 14 D along with dozens of downstream PGC-1α targets, particularly at 14 D (Table S1, Figure 1).

Surprisingly, genes involved in protein degradation as a group were not a prominent feature of the transcriptional profile of immobilization-induced muscle atrophy despite their putatively essential role in muscle atrophy in animal models [15]–[17]. Protein degradation genes, as a category, were only significantly up-regulated following 14 D of immobilization (Figure 1B). An examination of the genes contained within this category revealed a mix of Ub-conjugases, Ub-peptidases, 26S proteasome subunits, non-Ub proteases, and SUMOlating genes (Table S1). Importantly, atrogin-1 and MuRF-1, two Ub-conjugating enzymes that appear to have a crucial role in muscle atrophy [1], [13]–[17], were not among the genes in this category in any of the micro-array analyses. This suggests that protein degradation, as a category, is not a major aspect of the transcriptional program of human muscle atrophy and is only transcriptionally up-regulated during later stages of immobilization-induced atrophy.

### Validation of micro-array results using RT-PCR, western blotting and functional assays

In order to validate and extend the relationship between the atrophic transcriptome and functional alterations associated with atrophy, several different experimental approaches were applied. First, the mRNA level of selected genes was quantified using real time RT-PCR (qRT-PCR) (Figure S5). Significant changes in mRNA species encoding selected genes including PGC-1α, ATP synthase δ subunit, succinate dehydrogenase, hexokinase 2, and heme oxygenase-1 corroborated the results of the micro-array analyses (Figure S5). mRNA measurements using qRT-PCR were highly correlated (r^2^ = 0.996) with measurements obtained using gene micro-arrays over a large range of differential gene expression (20%–400%) (see Figure S5, inset frame). This indicates that the micro-array analyses provided highly sensitive and accurate, directional and quantitative measurements of changes in specific mRNA species.

**Figure 2:**
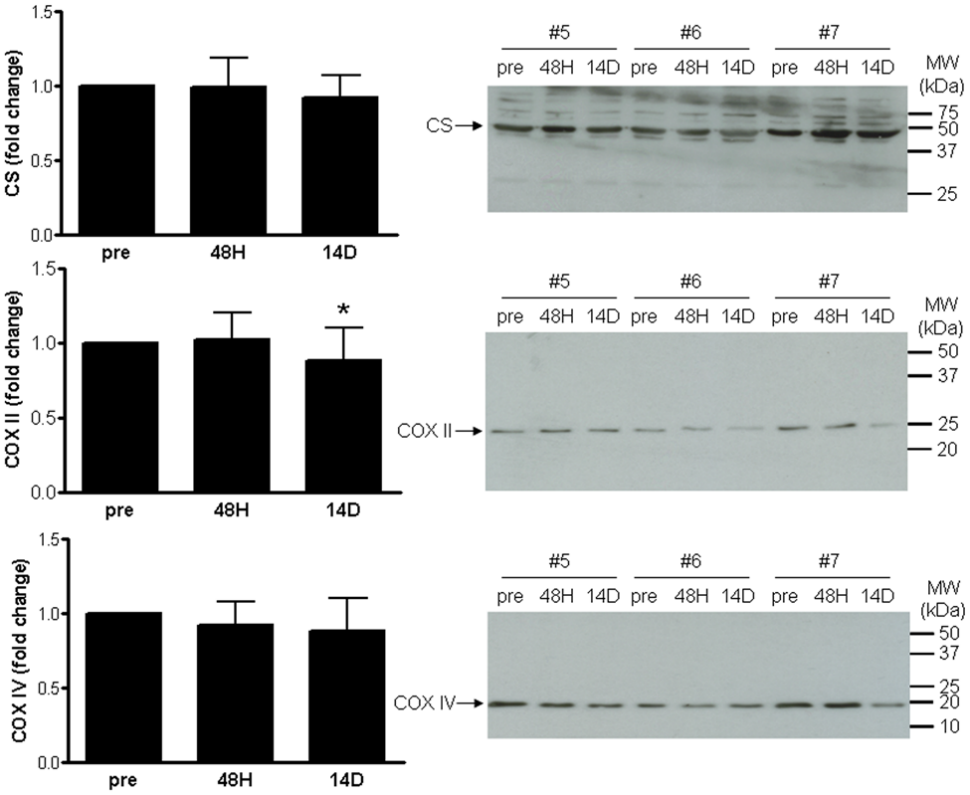
Changes in mitochondrial protein during immobilization-induced muscle atrophy. The protein level of the mitochondrial proteins citrate synthase (CS), cytochrome c oxidase (COX) subunits II and IV were determined by western blotting as detailed in the Materials and Methods. Protein levels were normalized to actin. Only COX II protein was significantly (P≤0.05) reduced following 14 D of immobilization (asterisk).

**Figure 3:**
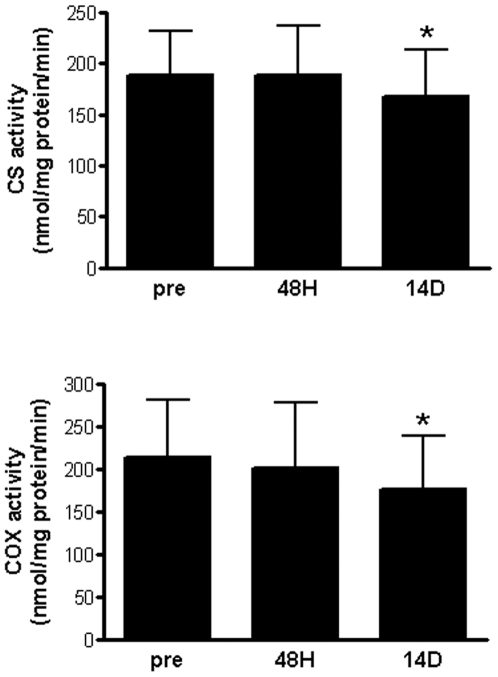
Changes in mitochondrial enzyme activity during immobilization-induced muscle atrophy. The enzyme activity of CS and COX was determined spectrophotometrically as detailed in the Materials and Methods. The enzymatic activity of both CS and COX were significantly (P≤0.05) reduced following 14 D of immobilization (asterisk).

Next, changes in mRNA abundance were evaluated at the protein level although limited sample prohibited the comprehensive evaluation of many of the transcriptional alterations. Thus a select group of proteins were examined to represent the primary theme that emerged regarding mitochondrial dysfunction (Table S1, Figures 1 and S5). The protein level of CS, an enzyme of the tricarboxylic acid (TCA) cycle usually taken to indicate total mitochondrial mass was not significantly reduced during the course of this study (Figure 2). The protein level of the electron transport chain (ETC) protein, COX subunit II (mitochondrial DNA encoded), was significantly decreased following 14 D of immobilization (P<0.01), while COX subunit IV (nuclear DNA-encoded) appeared to be similarly reduced but not significantly (Figure 2). Importantly, the maximal enzyme activity of both CS (P<0.005) and COX (P<0.01) decreased significantly by 14 D of immobilization (Figure 3). The extent of the decrease in COX subunit II protein corresponded closely to the decline in COX enzyme activity (both decreased by 18%). Thus, transcriptional down-regulation of several components of the TCA cycle and ETC by 14 D of immobilization is closely mirrored at the level of enzymatic function and is consistent with a general attenuation of mitochondrial bioenergetic function.

**Figure 4:**
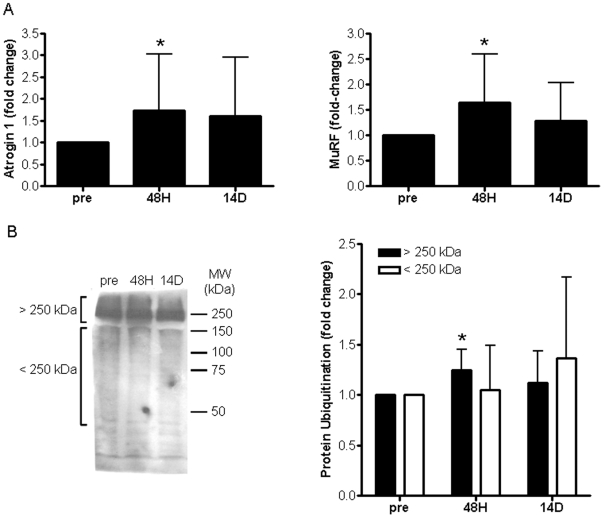
Transcriptional status of atrogin-1 and MuRF-1 and global protein ubiquitination during immobilization-induced muscle atrophy. (A) The mRNA level of both atrogin-1 and MuRF-1 was determined using qRT-PCR and significant (P≤0.05) increases in both genes were detected following 48 H of immobilization but not at 14 D. (B) Overall protein ubiquitination was examined using Western blotting and numerous ubiquitin-positive protein bands were detected (left). The most ubiquitin-dense proteins were greater than 250 kDa in MW. Thus lanes were divided into high MW (>250 kDa) and low MW (<250 kDa) sections and quantified using densitometry. A significant (P≤0.05) increase in protein ubiquitination of high MW proteins was detected following 48 H of immobilization (right, asterisk) but not 14 D. No significant changes in low molecular weight (<250 kDa) protein ubiquitination were detected at any time-point.

Due to the importance ascribed to atrogin-1 and MuRF-1 in muscle atrophy, the mRNA levels of these genes were targetted using qRT-PCR in spite of them not being identified as significantly changed by the micro-array bioinformatics approach. Figure 4A shows that the mRNA of both atrogin-1 and MuRF-1 increased (∼60%) significantly (P<0.05) following 48 H of immobilization. Although the mRNA of both these genes appeared elevated following 14 D of immobilization relative to pre-immobilization measures, this difference was not significant. To assess the implications of these findings at a functional level, overall protein ubiquitination was measured using Western blotting. To this end, muscle lysates were probed with an anti-Ub antibody and, as shown in Figure 4B, Ub-positive proteins were distributed over a large range of molecular weights with the most Ub dense signal appearing above 250 kDa. Individual lanes were divided into two groups (proteins weighing more or less than 250 kDa) and the Ub signal from each group was quantified using densitometry. A moderate (>20%), but significant (P<0.001) increase in the ubiquitination of high MW proteins was detected at 48 H following immobilization but not at 14 D (Figure 4B). No significant differences in the extent of ubiquitination of lower molecular weight proteins (<250 kDa) were detected at any time-point (Figure 4B). The co-temporal elevation of both MuRF-1 and atrogin-1 mRNA, and protein ubiquitination at 48 H supports some role for these atrogenes in the early stages of immobilization-induced muscle atrophy in humans; however our data does not support a quantitatively or coordinately robust induction of this program in short-term human immobilization induced muscle atrophy (Figure 4).

### Signaling pathways in immobilization-induced muscle atrophy

**Figure 5:**
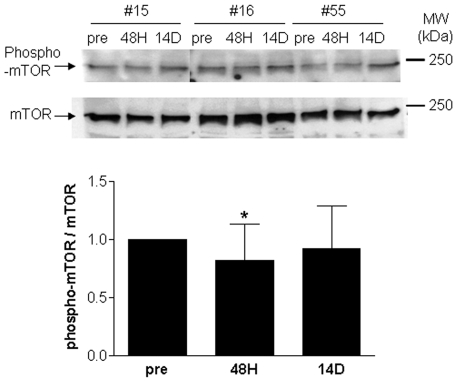
Changes in mTOR phosphorylation during immobilization-induced muscle atrophy. The protein level of phospho-mTOR was measured in muscle samples undergoing immobilization-induced muscle atrophy using Western blotting. Blots were then stripped and re-probed for total mTOR. Protein levels were normalized to tubulin. No significant differences in total mTOR protein levels were detected at any time-points (not shown), however the ratio of phospho-mTOR to mTOR decreased significantly (P≤0.05) at the 48 H time-point (asterisk) indicating decreased mTOR protein phosphorylation during the early phase of immobilization-induced muscle atrophy.

The major transcriptional theme to emerge from micro-array analysis of immobilized human muscle was a dramatic down-regulation of every aspect of mitochondrial structure and function. The mTOR signaling molecule has been shown to regulate mitochondrial biogenesis [42]. To examine the potential role of mTOR in human immobilization-induced muscle atrophy both the levels of total cellular mTOR and phospho-mTOR were measured and no significant change in the former was detected (not shown). However, the ratio of phospho-mTOR to total mTOR was significantly (P<0.05) reduced (by 20%) following 48 H but not 14 D of immobilization (Figure 5).

## Discussion

The global micro-array analysis conducted here represents an important step in defining the temporal pattern of the immobilization-induced atrophic transcriptome in human muscle. The most striking feature to emerge from the micro-array analyses is the dramatic over-representation of genes involved in metabolism and cell bioenergetics, particularly mitochondrial genes, within down-regulated transcripts (Figures 1 and S1). This pattern was observed as early as 48 H following immobilization and progressed through the duration of immobilization (14 D). In addition, genes that fall into the category of RNA synthesis and maturation were significantly up-regulated at both 48 H and 14 D (Figure 1), while genes that participate in protein synthesis were down-regulated at the later time-point (14 D) and genes that function in protein degradation were up-regulated at this time-point (Figure 1B). The transcriptional down-regulation of bioenergetic pathways is in general agreement with similar studies of muscle atrophy in murine models; however, genes involved in protein degradation were more prominent in the atrophic transcriptome of murine models, particularly in denervation based studies [16], [43], [44], Interestingly, an analysis of transcriptional changes that are specifically associated with the observed decline in muscle strength during immobilization did not yield any statistically significant genes (FDR q-value <0.05). This suggests that the decline in muscle strength is not mediated by changes in gene transcription within muscle itself, but may be mediated post-transcriptionally or by neuronal and/or hormonal adaptations to immobilization. Further work is needed to elucidate the underlying mechanisms that regulate changes in muscle strength following short-term immobilization, particularly, given the fact that women lost similar muscle mass and had an identical transcriptome response as compared to men, but had nearly twice the strength loss (Table 1) [32].

### Bioenergetics and metabolism

Virtually all facets of mitochondrial function including, oxidative phosphorylation, TCA cycle, fatty acid metabolism, mitochondrial transcription and translation, and solute and protein transport, were coordinately down-regulated (∼30% by 14 D, Table S1) at the transcriptional level by immobilization. These transcriptional changes were initiated very early during (48 H) the course of the transcriptional response to immobilization, indicating that mitochondrial bioenergetic pathways are rapidly targeted for transcriptional down-regulation following immobilization.

To examine the impact of the transcriptional suppression of mitochondrial genes, the protein levels and enzymatic activities of a subset of mitochondrial bioenergetic proteins were measured. Specifically, micro-array analysis revealed that CS mRNA was significantly down-regulated (∼20%) at 14 D and in association with atrophy (Table S1), and this was confirmed by qRT-PCR (PRE vs. 14 D) (Figure S5). Although CS protein expression was reduced over the course of immobilization, this reduction was not statistically significant (Figure 2). Similarly COX subunit IV (nuclear encoded) mRNA was significantly decreased (∼20%) at 14 D (micro-array and qRT-PCR, Table S1 and Figure S5), COX subunit IV protein levels were reduced at 14 D but not significantly (Figure 2). However, the enzyme activity of both CS and COX was significantly decreased by 14 D of immobilization (Figure 3). The concordance between mRNA and enzyme activity measures but not protein levels is likely a reflection of the higher variability of Western blotting compared to qRT-PCR and enzyme activity assays. In addition, COX subunit II (mitochondrial encoded) protein levels were significantly decreased (∼20%) following 14 D of immobilization indicating that the suppression of mitochondrial regulatory components (e.g. transcription/translation pathways) results in a detectable reduction in the expression of the COX subunit II mitochondrial protein.

Although other studies have reported decreases in the transcriptional and enzyme activity levels of specific components of muscle bioenergetic/mitochondrial systems after 1–6 weeks of unloading [45]–[48], few studies have examined global transcriptional patterns during muscle atrophy in humans [28], [30], [31], and only one has reported the large-scale suppression of mitochondrial genes during immobilization in humans [30]. However, only a small cohort (N = 4) was utilized and RNA samples were pooled from different subjects undergoing different periods of immobilization (4–11 d). Interestingly, the other study examined global transcriptional changes in humans following spinal cord injury and showed marked changes in the mRNA of protein degradation pathways [28]. This suggests that different transcriptional patterns are induced following denervation (spinal cord injury) and immobilization (casting/bracing).

This is the first report to show that the transcriptional down-regulation of mitochondrial genes comprises the predominant transcriptional feature of the atrophic transcriptome produced during short-term immobilization in humans. In addition, the large cohort used in this study enabled us to map out both early and late transcriptional patterns in human muscle during immobilization. The down-regulation of mitochondrial genes commenced as early as 48 H following immobilization. This pattern persisted over 14 D of immobilization and 73% of all significantly altered probes at 48 H were also altered at 14 D. Furthermore, this is the first study to demonstrate that the transcriptional suppression of mitochondrial genes is associated with reduced expression of mitochondrial proteins and decreased mitochondrial enzyme activity.

The evidence surrounding the involvement of mitochondrial dysfunction in muscle atrophy (induced by immobilization or denervation in animal models) has primarily focused on the contribution of mitochondrial dysfunction to increased oxidative stress and the induction of apoptosis [30], [49]–[51]. Results presented here indicate that mitochondria may participate in the atrophic response through their bioenergetic function. Direct support for this proposition comes from the observation that over-expression of PGC-1α in mouse skeletal muscle confers marked resistance to muscle atrophy following hindlimb unweighting [52], [53]. At present it is unclear how mitochondrial bioenergetics regulates muscle atrophy; however, alterations in mitochondrial bioenergetics are likely to influence other cellular pathways that are regulated by mitochondria, such as apoptosis, oxidative stress, and calcium signaling along with other ATP-dependent processes.

### Protein Synthesis and Degradation

Both protein synthesis and protein degradation are altered to varying degrees and at different time-points during human muscle atrophy. While animal studies have emphasized the importance of muscle protein degradation during muscle atrophy [1], [11], [13]–[17], the relative contribution of these processes to human muscle atrophy remains unclear. Early studies into the mechanism of muscle atrophy in humans concluded that the loss of muscle mass during bed rest was primarily due to the suppression of protein synthesis as muscle protein degradation was found not to change [23]–[25], [54]. Furthermore, the rate of myofibrillar protein synthesis was ∼50% lower in human muscle biopsy samples taken after 10 and 21 d of cast immobilization [26]. One study to date has measured the interstitial 3-methylhistidine (3-MH) concentration during immobilization-induced muscle atrophy in humans and demonstrated increased concentrations, presumably reflective of accelerated myofibrillar protein degradation at 72 h following ULS [27].

Results using micro-array analysis obtained here support a role for decreased protein synthesis in the later stages of human immobilization-induced muscle atrophy as genes encoding ribosomal subunits, translation initiation and elongation factors, and tRNA synthesizing enzymes were significantly down-regulated in the late (14 D) time-point of immobilization (Table S1, Figure 1B). Similarly, the micro-array analyses presented here indicate that protein degradation, as a functional category, is significantly up-regulated at a later (14 D) stage of immobilization-induced atrophy. This gene category is primarily comprised of components of the Ub-26S proteasome pathway, including Ub-conjugases, Ub-peptidases, subunits of the 26S proteasome, and SUMO-lating enzymes (Table S1). Surprisingly, neither atrogin-1 nor MuRF-1 were significantly altered in of the any micro-array analysis conducted despite being represented on the HU 133+ gene chips (Affymetrix). By contrast, atrogin-1 and MuRF-1 mRNAs were found to increase significantly at 48 H but not 14 D post-immobilization using qRT-PCR (Figure 4A). This discrepancy might be attributed to the high variance in the mRNA levels of these genes, which precluded their achieving statistical significance in the micro-array analyses. Importantly, a recent study indicated that increases in the mRNA levels of atrogin-1 and MuRF-1 was not accompanied by increased protein levels of these genes during denervation-induced muscle atrophy in humans [28]. That protein ubiquitination does play some role in the early phases of human muscle atrophy during immobilization is supported by the finding of a significant increase (∼20%) in the level of ubiquitination of high MW proteins (>250 kDa) at 48 H but not at 14 D (Figure 4B). While the specific identity of these ubiquitinated high MW proteins was not determined, MuRF-1 has previously been shown to participate in the ubiquitination of large cytoskeletal elements, including myosin heavy chain [12], [19], which are likely candidates for the high MW ubiquitinated proteins observed here. However, we were unable to detect atrogin-1 and MuRF protein using commercial antibodies (Abcam) and limited quantities of sample precluded direct measurements of Ub-proteasome activity. Thus, further work is needed before the increased protein ubiquitination observed here is attributed to MuRF-1 and/or atrogin-1 as well as to precisely define the role of proteasomal pathways in human immobilization-induced muscle atrophy.

### Signaling factors

A large number of signaling molecules and transcription factors were transcriptionally altered in all the micro-array analyses conducted (Table S1). However, categorical analysis of such factors is not useful because unlike components of metabolic pathways (e.g. oxidative phosphorylation), regulatory factors have highly context specific functions. In addition, the transcriptional regulation of such factors may nominally impact their functional status as other modes of regulation such as protein stability, phosphorylation status, binding partners, and sub-cellular localization can be more important determinants of functional activity. While the transcriptional status of such regulatory proteins cannot be used to ascertain their functional status, important clues can still be derived from the transcriptional status of down-stream functional genes. In particular, cluster analysis indicated that mitochondrial and bioenergetic genes cluster together which suggests that they are transcriptionally co-regulated. The results presented here indicate that PGC-1α mRNA is significantly down-regulated during both the early and late phases of immobilization-induced muscle atrophy (Table S1, Figure S5). In muscle, PGC-1α functions as a transcriptional co-activator that controls the expression of a large number of mitochondrial and metabolic genes [55]–[57]. Decreased PGC-1α mRNA along with the suppression of mitochondrial and metabolic genes may denote decreased PGC-1α activity during immobilization [57]. Although total PGC-1α protein content was not significantly altered by immobilization (not shown), PGC-1α protein level alone does not represent its functional status since phosphorylation, acetylation, and localization determine overall PGC-1α activity [52], [58].

Several cell signaling pathways have been implicated in muscle remodeling and mitochondrial biogenesis including Akt, NF-κB, and Ca^2+^-mediated signaling; however, the Akt signaling pathway is the best characterized regulator of both muscle hypertrophy and atrophy [59]-[69]. In the context of muscle atrophy, lower Akt activity decreases the activation of mammalian target of rapamycin (mTOR), leading to decreased protein synthesis and ribosomal biogenesis, as well as decreased mitochondrial biogenesis and function, as mTOR has been shown to regulate mitochondrial gene expression through the regulation of YY1/PGC-1α complex formation [42], [59], [60], [70]–[73]. In addition, lower Akt activity initiates signaling pathways that culminate in the induction of both atrogin-1 and MuRF-1 [14], [73], [74]. Phosphorylation of components of the Akt pathway was recently shown to decrease following 48 h of immobilization in humans [31]. Another study, however, failed to detect any significant changes in the phosphorylation of Akt, mTOR, p70^S6K^, eEF2, eIF4E, and TSC2 in men following 10 and 21 d of ULS [26]. Our examination found that while the ratio of phospho-Akt:Akt was not significantly altered following 48 H and 14 D of immobilization (not shown), the ratio of phospho-mTOR:mTOR decreased significantly following 48 H of immobilization but not 14 D of immobilization (Figure 5). Together, these findings suggest that signaling through mTOR may decline transiently during the early response of human muscle to immobilization and may contribute to decreased mitochondrial gene expression, possibly through interactions with PGC-1α [42].

In summary, the micro-array analyses presented here represent the most comprehensive evaluation of transcriptional changes during immobilization-induced muscle atrophy in humans. Our results highlight the importance of mitochondrial down-regulation, decreased mTOR phosphorylation, and increased protein ubiquitination during the early phases of the muscular response to immobilization in humans. The transcriptional profile during later phases of immobilization also show the coordinate down-regulation of every bioenergetic pathway, but are also marked by a reduction in protein synthesis pathways and an increase in some components of proteolytic systems. Much remains unclear with regard to the signaling mechanisms that precipitate the atrophic transcriptome in humans. Future work aimed at uncovering these mechanisms, beginning with an examination of the subcellular localization, post-translational modifications, and protein interactions of PGC-1α during muscle atrophy, could produce novel therapeutic strategies to combat pervasive pathological conditions associated with muscle atrophy in patients. In addition, future experiments focused on evaluating cellular proteasomal pathways by directly measuring the activity of Ub-proteasome, calpains, and caspases are vital to understanding the role of these systems in human muscle atrophy following immobilization.

## Supporting Information

Figure S1 Transcriptional changes associated with atrophy following immobilization. Simple linear regression trend tests of Log2 probe signal versus change in CSA were conducted to define transcriptional changes specifically associated with muscle atrophy. As in Figure 1, genes with an FDR q-value <0.05 were considered to be significantly associated with atrophy. Genes were then organized along functional lines and the number of genes in each category was plotted. Χ^2^ analyses were conducted on gene categories as in Figure 1 to determine whether gene categories were significantly (P≤0.05) up- or down-regulated in association with atrophy. Red bars are down-regulated genes whereas green bars are up-regulated genes. Arrowheads indicate gene categories that were significantly up- or down-regulated. Gene categories representing cellular bioenergetics, including fatty acid and carbohydrate metabolism and in particular mitochondrial function, were significantly down-regulated. Gene categories representing cytoskeletal components and regulators, oxidative stress, and metallothioneins were significantly up-regulated in association with atrophy. Significant alterations in the former two categories were unique to the analysis of atrophy-associated genes and were not significantly altered (as categories) in either the early (48H) or late (14D) analyses.
(0.01 MB PDF)

Figure S2 Cluster analysis of transcriptionally altered genes during the early (48H) phase of muscle atrophy. Genes whose transcription was significantly altered (FDR q-value <0.05) in two-time point comparisons early (48H) during the progression of immobilization-induced muscle atrophy were ordered into hierarchical clusters using HOPACH. HOPACH analysis produced 5 clusters, two of which (E1-1 and E1-2) represented up-regulated genes (A) and the remaining three (E2-1, E2-2, and E2-3) represented down-regulated genes (B). To determine the relationship between gene clusters and gene function, the distribution of genes (gene number) across the up-regulated and down-regulated clusters was plotted within each functional category. Χ^2^ analysis was employed to expose significant deviations between the actual number of genes within individual clusters in each functional category and the expected number of genes therein (detailed in the Materials and Methods). Asterisk indicates significant differences (P≤0.05) between the observed and expected partitioning of genes within clusters and functional categories.
(0.02 MB PDF)

Figure S3 Cluster analysis of transcriptionally altered genes during the late (14D) phase of muscle atrophy. Genes whose transcription was significantly altered (FDR q-value <0.05) in two-time point comparisons proximally (14D) during the progression of immobilization-induced muscle atrophy were ordered into hierarchical clusters using HOPACH. HOPACH analysis produced 11 clusters, six of which (L3-0, L4-1, L4-2, L4-3, L4-4, L4-5 and L4-6) represented up-regulated genes (A) and the remaining five (L1-0, L2-1, L2-2, L2-5, and L2-6) represented down-regulated genes (B). To determine the relationship between gene clusters and gene function, the distribution of genes (gene number) across the up-regulated and down-regulated clusters was plotted within each functional category. Χ^2^ analysis was employed to expose significant deviations between the actual number of genes within individual clusters in each functional category and the expected number of genes therein (detailed in the Materials and Methods). Asterisk indicates significant differences (P≤0.05) between the observed and expected partitioning of genes within clusters and functional categories.
(0.02 MB PDF)

Figure S4 Cluster analysis of atrophy-associated genes altered genes during immobilization-induced muscle atrophy. Genes whose transcription was significantly altered (FDR q-value <0.05) in trend tests of atrophy-associated genes (Log2 versus change in CSA) during immobilization-induced muscle atrophy were ordered into hierarchical clusters using HOPACH. HOPACH analysis produced 6 clusters, two of which (A1 and A2) represented up-regulated genes (A) and the remaining four (A3, A4, A5, and A6) represented down-regulated genes (B). To determine the relationship between gene clusters and gene function, the distribution of genes (gene number) across the up-regulated and down-regulated clusters was plotted within each functional category. Χ^2^ analysis was employed to expose significant deviations between the actual number of genes within individual clusters in each functional category and the expected number of genes therein (detailed in the Materials and Methods). Asterisk indicates significant differences (P≤0.05) between the observed and expected partitioning of genes within clusters and functional categories.
(0.02 MB PDF)

Figure S5 Quantitative real-time RT-PCR of selected genes at 48H (A) and 14D (B). The mRNA level of indicated genes was determined using quantitative real-time RT-PCR analyses (open bars) and compared directly to results from gene micro-array analyses (filled bars). Results obtained using both measures are plotted in the inset panels and the correlation coefficient was determined using linear regression. The high degree of correlation (R = 0.99) between mRNA measures obtained using two independent techniques (micro-array and RT-PCR) indicates the high accuracy of the micro-array analyses.
(0.02 MB PDF)

Table S1 Complete list of significantly altered genes from all analyses, organized by gene category.
(1.05 MB XLS)

Table S2 Genes altered by 48H but not following 14D of immobilization.
(0.04 MB XLS)
