# Supplementary figures and images for: Limb Immobilization Induces a Coordinate Down-Regulation of Mitochondrial and Other Metabolic Pathways in Men and Women
Source: PLoS One. 2009 Aug 5;4(8):e6518. doi: 10.1371/journal.pone.0006518 (PMC2716517; doi:10.1371/journal.pone.0006518)

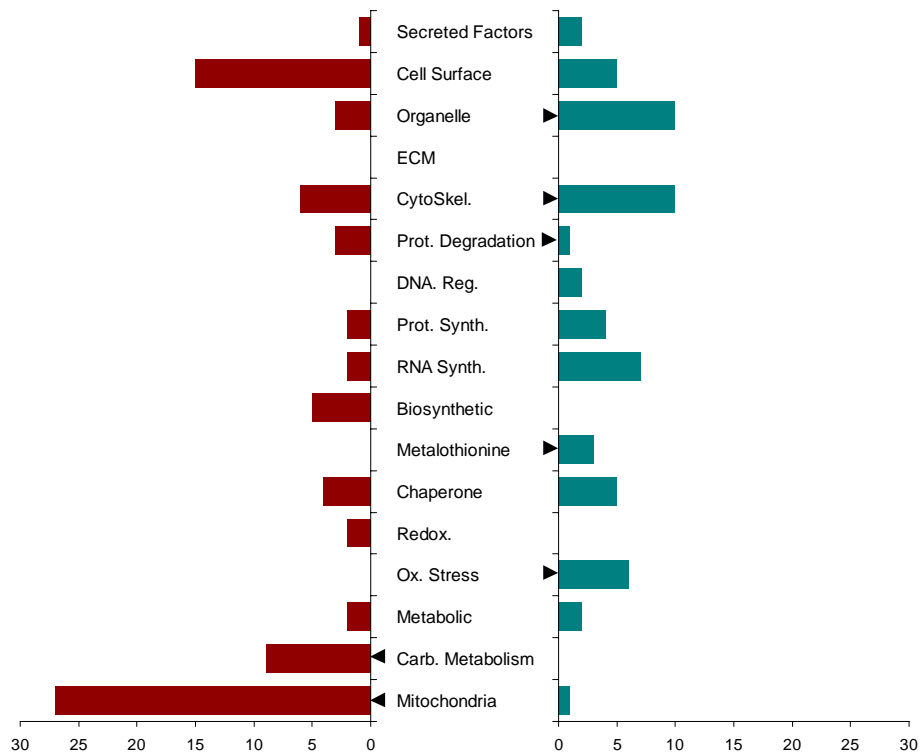

Supplement: Figure S1 — Transcriptional changes associated with atrophy following immobilization. Simple linear regression trend tests of Log2 probe signal versus change in CSA were conducted to define transcriptional changes specifically associated with muscle atrophy. As in Figure 1, genes with an FDR q-value <0.05 were considered to be significantly associated with atrophy. Genes were then organized along functional lines and the number of genes in each category was plotted. Χ2 analyses were conducted on gene categories as in Figure 1 to determine whether gene categories were significantly (P≤0.05) up- or down-regulated in association with atrophy. Red bars are down-regulated genes whereas green bars are up-regulated genes. Arrowheads indicate gene categories that were significantly up- or down-regulated. Gene categories representing cellular bioenergetics, including fatty acid and carbohydrate metabolism and in particular mitochondrial function, were significantly down-regulated. Gene categories representing cytoskeletal components and regulators, oxidative stress, and metallothioneins were significantly up-regulated in association with atrophy. Significant alterations in the former two categories were unique to the analysis of atrophy-associated genes and were not significantly altered (as categories) in either the early (48H) or late (14D) analyses. (0.01 MB PDF) [file pone.0006518.s001.pdf]

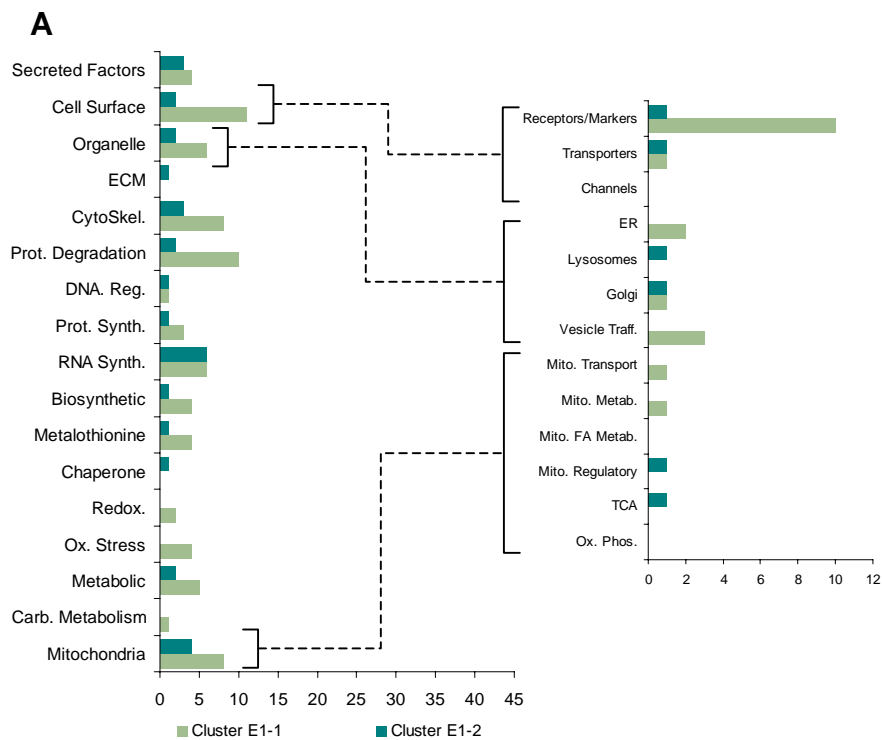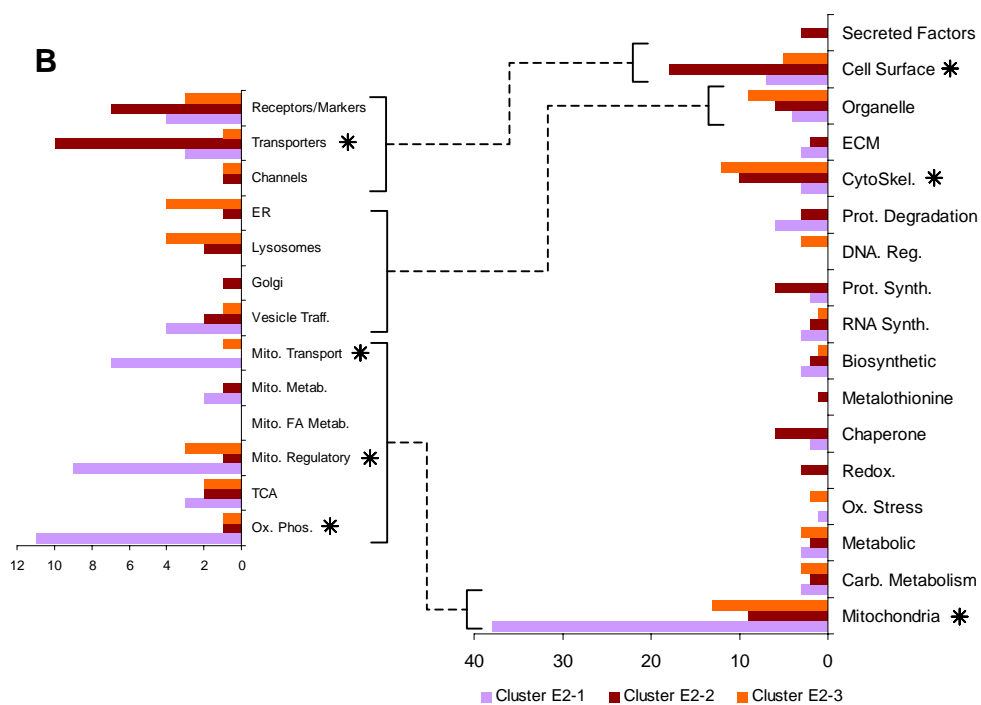

Supplement: Figure S2 — Cluster analysis of transcriptionally altered genes during the early (48H) phase of muscle atrophy. Genes whose transcription was significantly altered (FDR q-value <0.05) in two-time point comparisons early (48H) during the progression of immobilization-induced muscle atrophy were ordered into hierarchical clusters using HOPACH. HOPACH analysis produced 5 clusters, two of which (E1-1 and E1-2) represented up-regulated genes (A) and the remaining three (E2-1, E2-2, and E2-3) represented down-regulated genes (B). To determine the relationship between gene clusters and gene function, the distribution of genes (gene number) across the up-regulated and down-regulated clusters was plotted within each functional category. Χ2 analysis was employed to expose significant deviations between the actual number of genes within individual clusters in each functional category and the expected number of genes therein (detailed in the Materials and Methods). Asterisk indicates significant differences (P≤0.05) between the observed and expected partitioning of genes within clusters and functional categories. (0.02 MB PDF) [file pone.0006518.s002.pdf]

**A**

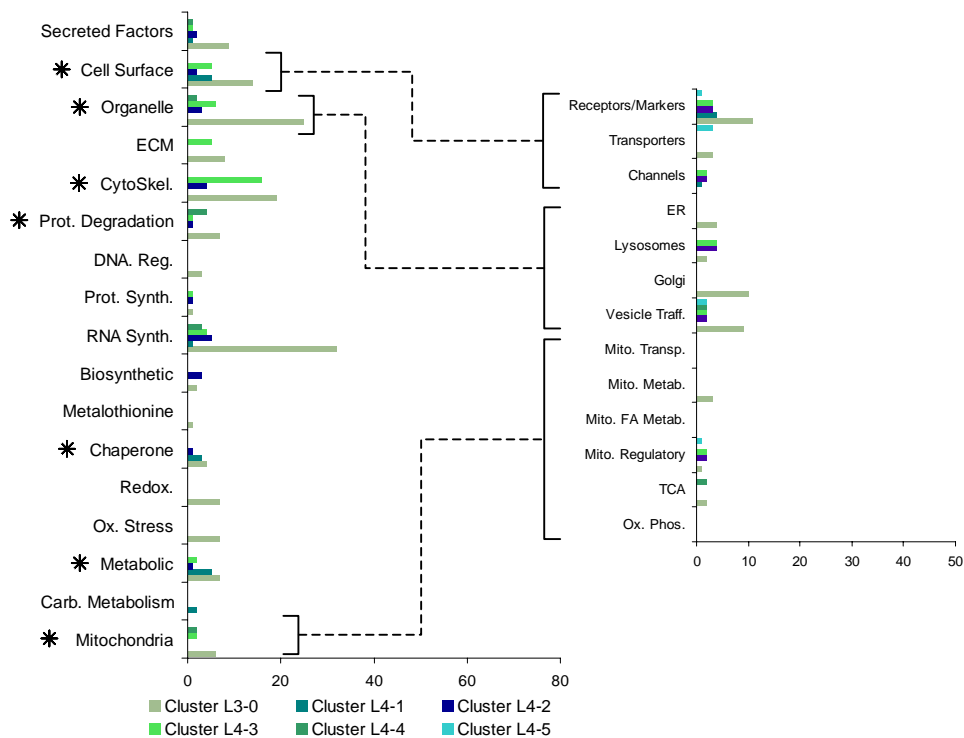

**B**

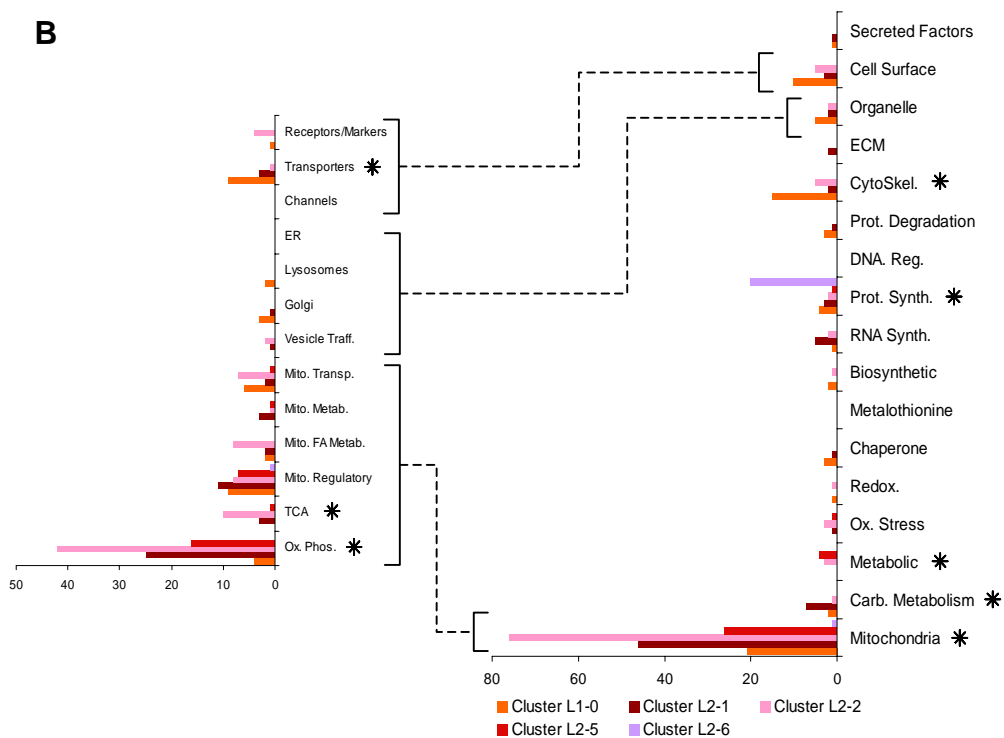

Supplement: Figure S3 — Cluster analysis of transcriptionally altered genes during the late (14D) phase of muscle atrophy. Genes whose transcription was significantly altered (FDR q-value <0.05) in two-time point comparisons proximally (14D) during the progression of immobilization-induced muscle atrophy were ordered into hierarchical clusters using HOPACH. HOPACH analysis produced 11 clusters, six of which (L3-0, L4-1, L4-2, L4-3, L4-4, L4-5 and L4-6) represented up-regulated genes (A) and the remaining five (L1-0, L2-1, L2-2, L2-5, and L2-6) represented down-regulated genes (B). To determine the relationship between gene clusters and gene function, the distribution of genes (gene number) across the up-regulated and down-regulated clusters was plotted within each functional category. Χ2 analysis was employed to expose significant deviations between the actual number of genes within individual clusters in each functional category and the expected number of genes therein (detailed in the Materials and Methods). Asterisk indicates significant differences (P≤0.05) between the observed and expected partitioning of genes within clusters and functional categories. (0.02 MB PDF) [file pone.0006518.s003.pdf]

**A**

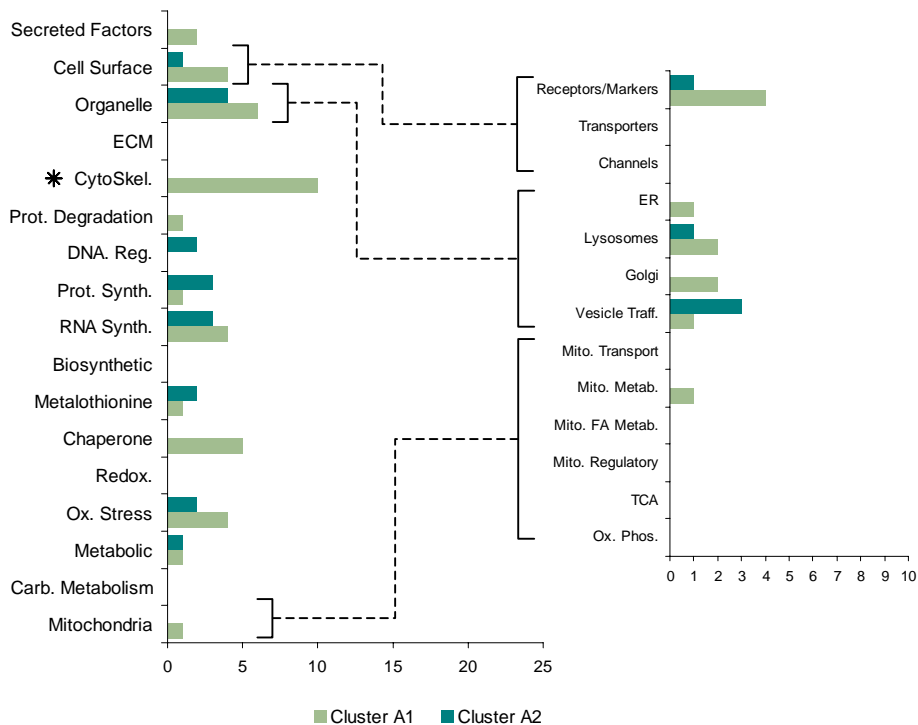

**B**

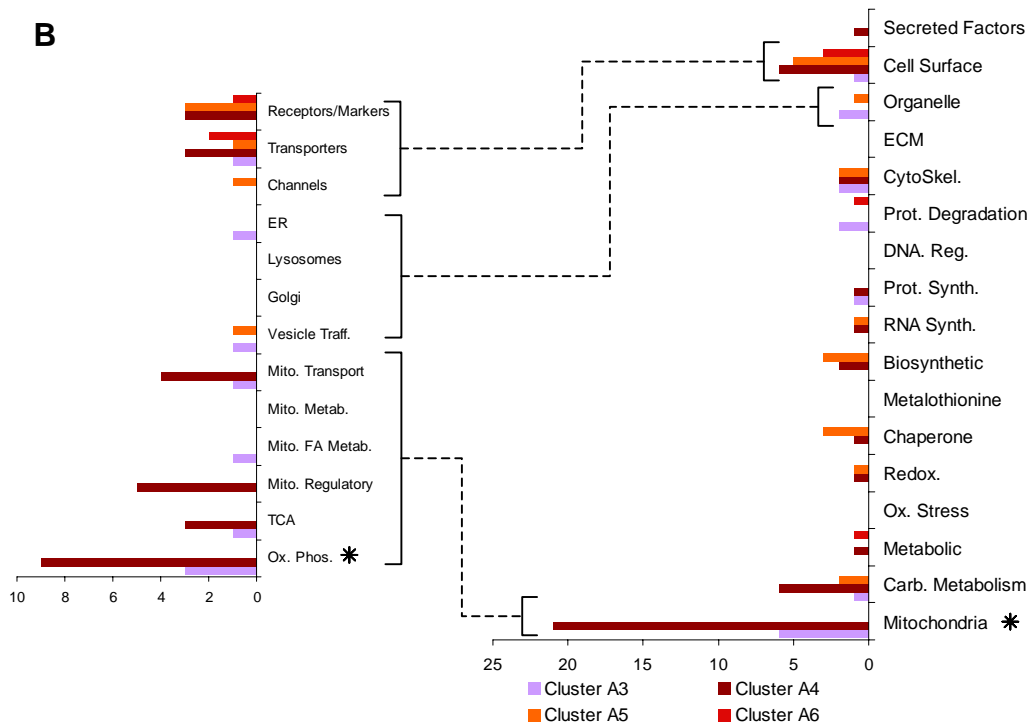

Supplement: Figure S4 — Cluster analysis of atrophy-associated genes altered genes during immobilization-induced muscle atrophy. Genes whose transcription was significantly altered (FDR q-value <0.05) in trend tests of atrophy-associated genes (Log2 versus change in CSA) during immobilization-induced muscle atrophy were ordered into hierarchical clusters using HOPACH. HOPACH analysis produced 6 clusters, two of which (A1 and A2) represented up-regulated genes (A) and the remaining four (A3, A4, A5, and A6) represented down-regulated genes (B). To determine the relationship between gene clusters and gene function, the distribution of genes (gene number) across the up-regulated and down-regulated clusters was plotted within each functional category. Χ2 analysis was employed to expose significant deviations between the actual number of genes within individual clusters in each functional category and the expected number of genes therein (detailed in the Materials and Methods). Asterisk indicates significant differences (P≤0.05) between the observed and expected partitioning of genes within clusters and functional categories. (0.02 MB PDF) [file pone.0006518.s004.pdf]

**A**

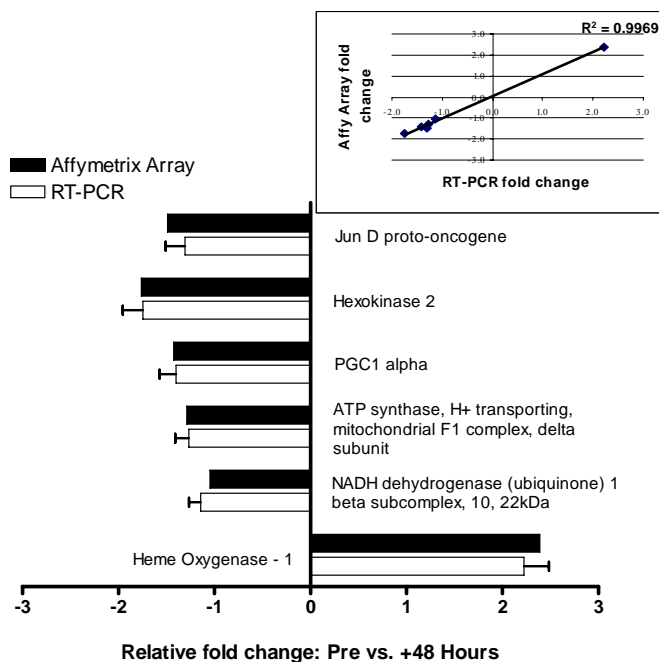

**B**

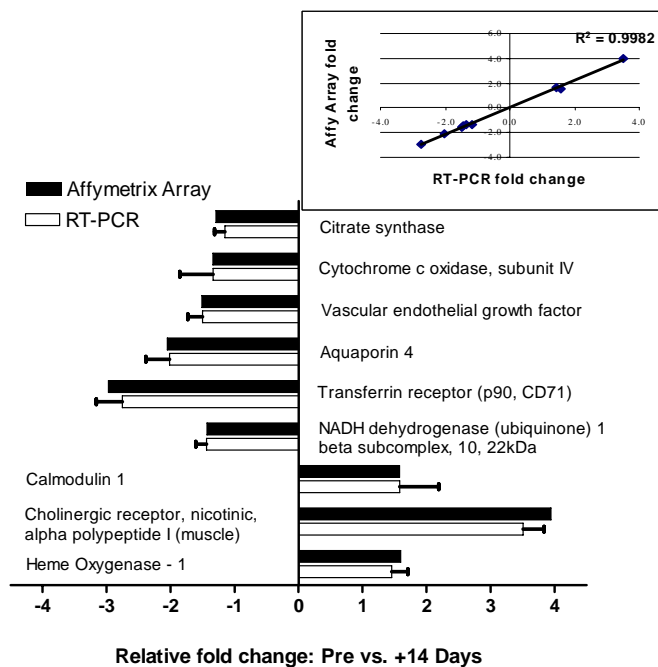

Supplement: Figure S5 — Quantitative real-time RT-PCR of selected genes at 48H (A) and 14D (B). The mRNA level of indicated genes was determined using quantitative real-time RT-PCR analyses (open bars) and compared directly to results from gene micro-array analyses (filled bars). Results obtained using both measures are plotted in the inset panels and the correlation coefficient was determined using linear regression. The high degree of correlation (R = 0.99) between mRNA measures obtained using two independent techniques (micro-array and RT-PCR) indicates the high accuracy of the micro-array analyses. (0.02 MB PDF) [file pone.0006518.s005.pdf]
